# Hexokinase 2 and Carbohydrate Metabolism: A Multifaceted Metabolic Hub

**DOI:** 10.3390/genes17070823

**Published:** 2026-07-19

**Authors:** Roman Maslanka, Justyna Folta, Magdalena Lubińska, Łukasz Słota, Renata Zadrag-Tecza

**Affiliations:** Department of Biochemistry and Cell Biology, Faculty of Biology, Nature Protection, and Sustainable Development, University of Rzeszów, 35-601 Rzeszów, Poland

**Keywords:** hexokinase 2, carbohydrate metabolism, metabolic regulation, carbon catabolite repression, gene expression, calorie restriction mimetic, mitochondrial bioenergetics, cellular physiology, reproductive potential, *Saccharomyces cerevisiae*

## Abstract

Hexokinase 2 (Hxk2p) is a key enzyme in glucose metabolism but also acts as a central regulator linking glucose-dependent signaling with cellular physiology in *Saccharomyces cerevisiae*. Beyond its catalytic function in glycolysis, Hxk2p acts as a regulator of carbon catabolite repression, influencing the expression of genes required for the utilization of alternative carbon sources and mitochondrial activity. Accumulating evidence indicates that deletion of *HXK2* triggers a systemic, multidirectional reprogramming of cellular metabolism and physiology that mimics calorie restriction conditions even in nutrient-rich environments. This widespread metabolic reconfiguration involves a fundamental shift from a rapid fermentative mode to an energy-efficient respiratory state, including the redistribution of carbon flux between glycolysis, the pentose phosphate pathway, and respiration. These changes are associated with alterations in ATP homeostasis, biosynthetic capacity, redox balance, and proteostasis. Crucially, because of its implications in genomic regulation, the absence of Hxk2p induces global transcriptional remodelling, whereby the expression of genes involved in respiratory and alternative carbon source metabolism is derepressed, while the expression of glycolytic and biosynthetic genes is downregulated. Ultimately, these pleiotropic adaptations work synergistically, affecting cellular fitness and increasing cell reproductive potential. Therefore, Hxk2p integrates metabolic and signaling pathways that link carbon source utilization with cellular growth, cell cycle, energy homeostasis, and stress responses. This review summarizes current knowledge on Hxk2p function in carbohydrate metabolism, with particular emphasis on its regulatory roles and their implications for gene expression, cellular physiology, and proliferation capacity.

## 1. Introduction

The budding yeast *Saccharomyces cerevisiae* has long been an important model for understanding the genetic and metabolic basis of maintaining cell physiology and cell aging. Its glucose-based and well-characterized central carbon metabolism provides not only energy but also the essential building blocks for cellular macromolecules. Numerous studies have shown that glucose acts as a potent signaling molecule, coordinating metabolism, growth, cell size, proliferation, and stress responses through interconnected cellular pathways. Within this network, hexokinase 2 (Hxk2p) appears to be a multifaceted metabolic hub that bridges primary catabolic flux with global transcriptional regulation, physiological efficiency of cells, and their ability to proliferate. This concept forms the basis of the present review, which also incorporates findings from our previous experimental studies. This review is a narrative synthesis focusing primarily on hexokinase 2 (Hxk2p) in the budding yeast *S. cerevisiae*. Peer-reviewed publications addressing both the catalytic activity and regulatory functions of Hxk2p were considered, regardless of their date of publication. However, particular emphasis was placed on recent findings (published between 2010 and 2026) that describe the pleiotropic consequences of *HXK2* deletion and their implications for systemic and multidirectional aspects of cellular physiology. The literature cited in this work was selected through a comprehensive search of major scientific databases, including PubMed, Web of Science, Scopus, and SGD, utilizing keywords such as ‘hexokinase 2’, ‘Hxk2 AND yeast’, ‘*HXK2* AND *Saccharomyces cerevisiae*’, ‘hexokinase 2 AND *Saccharomyces cerevisiae*’, ‘Hxk2 AND carbon catabolite repression’, ‘Hxk2 AND glucose metabolism’, ‘*Saccharomyces cerevisiae* AND glucose metabolism’, ‘yeast AND carbohydrate metabolism’, ‘*HXK2* AND metabolic regulation’, ‘*HXK2* AND cellular physiology’, ‘*HXK2* AND replicative lifespan’, ‘*HXK2* AND gene expression’, ‘*HXK2* AND metabolic flux’, ‘*HXK2* AND respiration’, ‘*HXK2* AND mitochondrial activity’, ‘*HXK2* AND cellular energy’, ‘*HXK2* AND metabolic reprogramming’, ‘*HXK2* AND glycolysis’, ‘*HXK2* AND hexose transporters’, ‘*HXK2* AND glucose signaling’, ‘*HXK2* AND carbon flux’, ‘*HXK2* AND redox balance’, ‘*HXK2* AND biosynthesis’, ‘*HXK2* AND oxidative stress’, ‘*HXK2* AND pentose phosphate pathway’, ‘*HXK2* AND proteostasis’, ‘*HXK2* AND stress response’, ‘*HXK2* AND nuclear localization’, ‘*HXK2* AND moonlighting protein’, ‘*HXK2* AND epigenetics’, ‘*HXK2* AND cell size’, ‘*HXK2* AND growth’. Additional relevant publications were identified by screening the reference lists of selected articles. Specifically, we summarize current knowledge of both the canonical and noncanonical functions of yeast Hxk2p and discuss how deletion of the *HXK2* gene triggers a systemic and multidirectional reprogramming of the transcriptome, metabolism, and cellular physiology. Our current understanding of the Δ*hxk2* phenotype is largely based on our previous experimental studies [1,2,3], which demonstrate that the absence of Hxk2p drives cells into a distinct metabolic loop, redirecting carbon resources from biomass production toward cellular energy maintenance. Consequently, the enhanced reproductive potential of Δ*hxk2* cells results from a systemic reprogramming of metabolic fluxes rather than increased respiratory activity alone. Remarkably, many of these changes generate a phenotype that mimics caloric restriction (CR) conditions, despite the presence of abundant glucose.

## 2. Yeast Carbohydrate Metabolism, Its Regulatory Dynamics and Physiological Significance

Central carbon metabolism (CCM) represents the core axis of cellular processes and plays a fundamental role in maintaining cellular physiology. CCM not only provides energy generation but also the carbon skeletons necessary for the biosynthesis of essential macromolecules, including nucleotides, amino acids, and lipids [4,5,6,7,8,9]. In most eukaryotic cells, including the budding yeast *S. cerevisiae*, this system is predominantly based on glucose, which is considered a primary source of energy. However, as more and more research shows, the role of glucose extends far beyond its function as a simple cellular fuel; it acts as a potent signaling molecule [4,10,11,12,13,14,15]. Glucose availability impacts cellular physiology by modulating levels of intracellular metabolites, enzymatic activities, mRNA stability, and global gene expression profiles [1,2,4,10,11,14,16,17,18,19]. These signaling activities mainly involve interconnected pathways: the Snf1/Mig1/Hxk2 pathway—responsible for carbon catabolite repression; the Snf3/Rgt2 pathway—responsible for extracellular glucose sensing and the expression of hexose transporter (*HXT*) genes; the Ras/cAMP/PKA pathway—responsible for growth, proliferation, and stress response coordination [4,10,11,12,13,14,15,19,20,21,22,23,24,25,26,27]. The Snf1/Mig1/Hxk2 pathway enables yeast cells to preferentially utilize glucose by repressing genes involved in respiration and alternative carbon-source metabolism. Under glucose-rich conditions, Hxk2p cooperates with Mig1p/Mig2p to form the repressor complex on target promoters and repress gene expression. Upon glucose depletion, active Snf1 kinase phosphorylates both Hxk2p and Mig1p, resulting in the disassembly of the repressor complex and derepression of target genes [11,14,19,20,21,28,29,30,31,32]. The Snf3/Rgt2 pathway enables cells to sense extracellular glucose concentrations and adjust glucose uptake. Snf3p and Rgt2p, although structurally related to glucose transporters, evolved as specific glucose sensors. Rgt2p and Snf3p are activated by different levels of glucose; however, activating either of them promotes degradation of the Mth1p and Std1p repressors, leading to Rgt1p phosphorylation, its dissociation from Ssn6p-Tup1p, and consequently to the induced expression of hexose transporter (*HXT*) genes [4,11,12,14,15,22,25,33,34,35,36,37]. Rgt2p detects high levels of glucose and stimulates the expression of low-affinity glucose transporters (e.g., *HXT1* and *HXT3*). In turn, Snf3p detects low levels of glucose and stimulates the expression of high-affinity and moderate-affinity glucose transporters (e.g., *HXT2*, *HXT4*, and *HXT6*) [11,14,34,37,38,39]. The Ras/cAMP/PKA pathway is one of the primary signalling pathways that not only coordinates growth, metabolism, proliferation, and stress responses in yeast cells but also adjusts cell metabolism to the current nutrient status. In the presence of glucose, high activity of the cAMP/PKA pathway stimulates growth-associated processes, including glycolysis and ribosome biogenesis, while simultaneously suppressing respiratory metabolism and stress-response mechanisms [1,4,12,14,23,26,27,40,41,42]. Activation of this pathway occurs through both extracellular glucose sensing connected with the G-protein-coupled receptor (GPCR) system and intracellular glucose metabolism connected with activation of Ras proteins [4,42,43,44,45]. The GPCR system is composed of the plasma membrane receptor Gpr1p and its α-subunit of the G-protein Gpa2p. Ultimately, both pathways lead to activation of adenylate cyclase (Cyr1p) and synthesis of cAMP, which directly activates PKA (Protein Kinase A) [14,27,42,43,45,46]. Since glucose is a metabolic substrate and also acts as a metabolism-modulating molecule, its availability must be constantly monitored, and changes in its concentration may induce different modes of its intracellular use. The integration of environmental and metabolic signals ensures that cell growth and proliferation are precisely coupled with the current nutritional status [1,3,4,7,14,23,47,48,49,50]. Carbohydrate metabolism includes a complex network of interrelated metabolic pathways: glycolysis, the pentose phosphate pathway (PPP), and respiration with mitochondrial oxidative phosphorylation (OXPHOS). The cooperation between these routes provides a continuous supply of energy (ATP), biosynthetic precursors, cofactors, and reducing equivalents (e.g., NAD(P)H) for enzyme activities and redox homeostasis [1,3,4,5,51,52,53,54]. Therefore, metabolic flux between these pathways is dynamically adjusted to fluctuating extracellular conditions and actual intracellular demands. This specific “cellular economics” allows cells to optimize their growth rates and adapt their metabolism and survival strategies to stress conditions [1,23,47,49,52,53,55,56,57,58,59]. A defining feature of the budding yeast *S. cerevisiae* is its preference for aerobic glycolysis and fermentation over the more energy-efficient respiratory metabolism, even in the presence of large amounts of oxygen (the Crabtree effect). It is currently postulated that this metabolic strategy enables rapid ATP production while providing abundant carbon precursors for biosynthetic processes and generating ethanol, which inhibits the growth of competing microorganisms [23,49,60,61,62,63,64,65,66,67,68]. This metabolic preference is driven by glucose-mediated repression of genes required for respiratory activity and is initiated at the first steps of glucose utilization.

## 3. Hexokinases in Yeast: Catalytic Activity and Regulatory Functions

The rate of glucose/hexose uptake and the efficiency of its initial catabolic steps markedly determine the overall glycolytic flux and glucose-dependent intracellular signaling [1,2,3,9,12,14,50,69,70,71,72,73]. Consequently, hexokinases, which catalyze the first irreversible step of glucose metabolism, may function as key regulatory gatekeepers linking extracellular nutrient availability with the intracellular metabolic state. Hexokinases (HXKs) are a fundamental class of enzymes with kinase activity, catalyzing the ATP-dependent phosphorylation of glucose at the C6 position to produce glucose-6-phosphate (G6P). G6P is a membrane-impermeable form, directing carbon flux into downstream metabolic routes such as glycolysis, the pentose phosphate pathway (PPP), and trehalose synthesis [3,9,14,69,74]. Yeast *S. cerevisiae* possesses three hexokinase isoenzymes: hexokinase 1 (Hxk1p; encoded by the *HXK1* gene), hexokinase 2 (Hxk2p; encoded by the *HXK2* gene), and glucokinase (Glk1p; encoded by the *GLK1* gene). Although all three enzymes support growth on glucose, they differ in their biochemical characteristics and physiological functions. Hxk1p and Hxk2p are closely related paralogs sharing approximately 77% amino acid identity and show broad substrate specificity. They can phosphorylate both aldo- and ketohexoses, including glucose, fructose, and mannose. In contrast, Glk1p is specific for aldohexoses, exhibiting lower sequence similarity (37% identity). Structurally, yeast hexokinases are monomers of approximately 54 kDa; however, both Hxk1p and Hxk2p are also capable of forming native ~110 kDa homodimers [9,75,76,77,78,79,80,81,82,83,84]. During growth on glucose, Hxk2p is the predominant isoenzyme, which, besides acting as a glycolytic enzyme, is identified as a “moonlighting” protein with non-metabolic regulatory functions. Among others, Hxk2p is involved in intracellular signaling, sensing of intracellular glucose levels, and the coordination of carbon catabolite repression (CCR). In the presence of glucose, a fraction of Hxk2p (approximately 15%) translocates into the nucleus [19,28,69,77,78,82,83,85,86,87,88]. There, in cooperation with the transcriptional repressors Mig1p and Mig2p, the kinase Snf1p, and the phosphatase Reg1p, it forms a repressor complex that binds to the promoters of several genes. The repressed genes include genes required for the utilization of alternative carbon sources (e.g., *SUC2*—invertase gene; *GAL* genes—galactose metabolism genes; *MAL* genes—maltose metabolism genes), high-affinity glucose transporter genes (e.g., *HXK6*, *HXT7*), and genes responsible for mitochondrial activity and respiratory metabolism [11,14,15,19,20,21,28,32,89]. Moreover, Hxk2p inhibits the expression of its own paralogs, the *HXK1* and *GLK1* genes, ensuring that under conditions of high glucose levels, Hxk2p remains the primary kinase and plays an important role in the coordination of cell metabolic priorities with nutrient availability [19,84]. Consequently, the classical view of yeast Hxk2p as a simple glycolytic enzyme has evolved toward recognizing it as a multifaceted metabolic hub that links central carbon metabolism with global gene expression, energy metabolism, biosynthetic capacity, cellular growth strategies, and cell reproductive potential [1,2,3,18,19,30,88,90,91,92].

## 4. Hexokinase 2 as a Multifaceted Metabolic Hub: Pleiotropic Consequences of *HXK2* Deletion for Cellular Physiology

As a metabolic hub, Hxk2p integrates carbon flux with global regulatory networks. Consequently, deletion of the *HXK2* gene induces extensive metabolic and transcriptional remodeling, generating physiological and molecular features of a calorie restriction-like (CR-like) phenotype even under nutrient-rich conditions. Therefore, the Δ*hxk2* strain is regarded as a genetic model that mimics CR [2,3,93,94,95,96]. Deletion of *HXK2* reorganizes cellular resource allocation, shifting the cellular strategy from rapid biomass production and fermentative growth toward an energy-efficient, stress-resistant respiratory state [1,3,97,98,99]. The major hallmarks of this remodeling include attenuation of the Crabtree effect, elevated intracellular ATP levels, reduced oxidative burden, and global downregulation of energetically costly biosynthetic processes. These adaptations work synergistically to decrease cell size, reduce anabolic burden, improve proteostasis, and enhance protein quality control, thereby supporting the maintenance of physiological homeostasis and increasing the reproductive potential of the cell [1,2,3,97,98,100,101,102]. The multifaceted nature of these effects is summarized in Table 1, Table 2, Table 3 and Table 4, which categorize the pleiotropic consequences of *HXK2* deletion across various levels of cellular organization, ranging from gene expression and metabolic regulation to physiological and morphological features.

### 4.1. Loss of Carbon Catabolite Repression (CCR) Following HXK2 Deletion

One of the most pronounced consequences of *HXK2* deletion in *S. cerevisiae* is the disruption of CCR, resulting in large-scale derepression of genes normally repressed by high glucose levels. The absence of Hxk2p prevents the assembly of the Mig1p-dependent nuclear repressor complex, which allows for the phosphorylation of Mig1p by the constitutively active Snf1p kinase and the subsequent export of this repressor to the cytoplasm [1,3,11,14,19,20,21,28,98,99,100,103,104,105,106]. At the transcriptional level, this is manifested through enhanced expression of genes such as *SUC2* and those required for utilizing alternative carbon sources, like the *GAL* and *MAL* genes. Importantly, *HXK2* deletion also leads to derepression of genes associated with respiratory metabolism, including genes involved in the tricarboxylic acid (TCA) cycle, electron transport chain (ETC), and ATP synthase formation, such as *CIT1*, *IDH1/2*, *SDH1/2/4*, *NDI1*, *CYT1*, *COX2*, *ATP2*, and *ATP3*. This promotes a shift from fermentative to respiratory metabolism even under high-glucose conditions, which is manifested by a significant increase in intracellular ATP levels, attenuation or abolition of the Crabtree effect, and the establishment of a “respiratory-primed” cellular state [1,3,14,19,89,98,99,100,103,105,107,108]. Furthermore, the Δ*hxk2* strain exhibits relevant remodeling of the hexose transporter system, characterized by the downregulation of low-affinity transporters (*HXT1*, *HXT3*) and concomitant induction of medium- and high-affinity transporters (*HXT2*, *HXT4*, *HXT6*, *HXT7*) [1,14,99,103,109]. *HXK2* deletion also causes increased expression of the *HXK1* and *GLK1* genes, which normally play a secondary role in glucose phosphorylation and whose activity compensates for the loss of Hxk2p catalytic activity [1,14,19,84,105]. Together, these pleiotropic changes result in expanded carbon source utilization, loss of hierarchical utilization of sugars, the ability to simultaneously utilize various sugars and ethanol, elimination of adjustment phases during nutrient shifts, and metabolic adaptation of cells to conditions mimicking low glucose concentration—CR (Table 1).

### 4.2. Remodeling Carbohydrate Metabolism and Central Carbon Flux

The deletion of the *HXK2* gene induces relevant remodeling of the hexose transporter system and carbohydrate utilization, the direct effects of which are a significant reduction in the rate of glucose uptake, decreased utilization of simple carbohydrates, and decreased overall glycolytic flux [1,2,3,19,97,100,106]. At the metabolic level, the Δ*hxk2* strain shows altered dynamics of glycolytic intermediates, most notably increased glucose-6-phosphate (G6P) and decreased fructose-1,6-bisphosphate (F1,6bP) levels. The elevated G6P/F1,6bP ratio may act as a metabolic switch that redirects carbon flux from fermentation toward the TCA cycle and mitochondrial respiration, thereby abolishing the Crabtree effect [1,3,73,84,97,100,104,110]. This metabolic shift is consistent with reduced ethanol production, lowered activity of pyruvate decarboxylase, and intracellular pyruvate accumulation. Additionally, it has been shown that the Δ*hxk2* strain can co-consume glucose and ethanol simultaneously [2,97,99,100,104,105,106]. Furthermore, deletion of the *HXK2* gene also alters pentose phosphate pathway (PPP) activity and NADPH metabolism. Reduced activity of PPP enzymes, together with lower NADPH consumption, appears to be associated with a decreased biosynthetic burden and a redirection of PPP-derived metabolites toward de novo nucleotide synthesis rather than the production of other cellular macromolecules [1,3]. Ultimately, the redirection of carbon flux from fermentation toward respiratory metabolism and the alterations in PPP activity are also associated with decreased cAMP levels and reduced activity of the Ras/cAMP/PKA pathway [1,3,14,23,105,106,111]. Moreover, recent work showed that beyond the Snf1/Mig1 and cAMP/PKA axes, glucose metabolism also acts as a direct trigger for the Target of Rapamycin Complex 1 (TORC1), the crucial regulator of eukaryotic cell growth and proliferation. Studies demonstrated that *S. cerevisiae* employs three distinct pathways to link glycolysis with TORC1 activity: (i) a canonical Rag GTPase-dependent pathway requiring F1,6bP, (ii) a non-canonical pathway requiring G6P and mitochondrial function, and (iii) a Rag-independent pathway requiring complete glycolysis and V-ATPase reassembly. However, regardless of the pathway, hexokinase-mediated glucose phosphorylation is the indispensable ‘gatekeeping’ step for TORC1 activity. Consequently, hexokinase-deficient cells fail to activate TORC1 in response to glucose, thereby uncoupling nutrient availability from anabolic processes and cell growth [112]. This results in reduced ribosome biogenesis and protein synthesis, thereby lowering the anabolic burden, promoting a small-cell phenotype and a state resembling CR (Table 1).

**Table 1:** Regulation of carbon catabolite repression and systemic remodeling of central carbon flux in *HXK2* gene deletion and the absence of Hxk2p.

Functional Level/ Effect Category	Parameter	Effect of *HXK2* Gene Deletion	Physiological Consequence	References
**Carbon catabolite** **repression (CCR)**	Global glucose repression	Derepression of a wide range of glucose-inhibited genes, including mitochondrial, respiratory, hexose transporter and alternative carbon utilisation genes	Shift from fermentative to respiratory metabolism; attenuation/elimination of the Crabtree effect; expanded capacity to utilize different carbon sources	[1,3,13,14,19,28,84,99,100,104,105,106]
	Hxk2p–Mig1p nuclear repressor complex	Lack of Hxk2p sequestration in the nucleus; Lack of physical Hxk2p–Mig1p interaction; Enabling Mig1p phosphorylation by Snf1p; Induction of Mig1p repressor export from the nucleus to the cytoplasm	Impaired nuclear repression complex formation; lack of interaction of the Mig1p-dependent repressor complex with target gene promoters; removal of the transcriptional blockade of glucose-repressed genes	[14,19,20,21,28,103]
	Sucrose and alternative carbon metabolism	Derepression of the *SUC2* gene (encoding invertase), active transcription of galactose metabolism (*GAL* genes) and maltose metabolism (*MAL* genes); Expression of maltose transporters and gluconeogenesis enzyme genes	Possibility of simultaneous hydrolysis of sucrose or utilization of alternative carbon sources (e.g., galactose, maltose) even in the presence of glucose; loss of hierarchical utilization of sugars	[14,19,89,98,99,100,105,108]
	Regulation of hexokinase isoenzyme expression	Derepression and strong induction of *HXK1* and *GLK1* gene expression even under high glucose conditions	Compensation of glucose phosphorylation activity by alternative hexokinases (Hxk1 and Glk1); alteration of intracellular glucose phosphorylation level and glucose signaling; conditions mimicking low glucose concentrations and adaptation to them	[1,14,19,84,87,100,105]
	Respiratory metabolism and mitochondrial biogenesis repression	Induced expression of TCA cycle genes (e.g., *CIT1*, *IDH1/2*, *SDH1/2/3/4*), ETC—electron transport chain genes (e.g., *NDI1*, *CYT1*, *COX4*, *COX6*, *COX2*), ATP synthase genes (e.g., *ATP1*, *ATP2*, *ATP15*, *ATP17*) and components of the mitochondrial translation machinery	Increased activity of oxidative metabolism; development of the mitochondrial network and increased ATP production; decoupling of mitochondrial biogenesis from glucose availability	[1,3,99,100,103,105,107]
**Carbohydrate** **metabolism**	Hexose transporters (*HXTs*) expression and trafficking	Decrease in gene transcription and protein levels of low-affinity transporters (*HXT1*, *HXT3*); Derepression and increased expression of medium- and high-affinity transporters (*HXT2*, *HXT4*, *HXT6*, *HXT7*)	Modified glucose uptake dynamics; altered glucose-dependent intracellular signaling; metabolic adaptation of cells to conditions mimicking low glucose concentrations—calorie restriction (CR)	[1,14,99,103,109]
	Rate of glucose uptake and carbohydrate utilization	Significantly reduced glucose uptake (measured with the 6-NBDG analogue); Significantly slower rate of utilization of simple carbohydrates (both glucose and fructose) from the medium	Altered intracellular glucose levels; decreased glycolytic flux and reduced fermentative capacity; shift of metabolism towards mitochondrial respiration	[1,2,3,100,106]
	Glucose phosphorylation and glycolytic intermediates dynamics (glucose-6-phosphate (G6P); fructose-1,6-bisphosphate (F1,6bP); pyruvate)	Altered hexokinase phosphorylation activity (compensated by *HXK1* and *GLK1* gene depression); Increased G6P level; Decreased F1,6bP level; Intracellular pyruvate accumulation	Remodeling of metabolic profile and flux; high G6P/F1,6bP ratio (abolishes the Crabtree effect and stimulates respiration); decreased F1,6bP-dependent allosteric activation of pyruvate kinase and redirection of carbon flux to the TCA cycle	[1,3,73,84,97,100,104,110]
	cAMP/PKA signaling activity	Reduced cAMP levels; Attenuated protein kinase A (PKA) activity resulting from altered glucose signaling	Reduction of glycolytic flux; derepression of respiratory metabolism genes; reduction of ribosome biogenesis and protein biosynthesis energy expenditures; activation of stress response mechanisms	[1,3,14,23,111]
	TORC1 signaling activity	Disrupted glucose phosphorylation; Altered levels of glycolytic intermediates (G6P, FBP); Disrupted glucose-induced TORC1 activation pathways	Disruption of the link between glycolysis and TORC1 activity; altered regulation of cell growth; reduced anabolic stimulation (protein/lipid synthesis) and potentially enhanced catabolic processes (autophagy/proteasomal activity)	[112]
**Central carbon flux**	Glycolytic flux	Decreased flux through glycolytic enzymes; Downregulated expression of genes associated with glycolysis (e.g., *GPM1*, *ENO2*); Decreased activity of glycolytic and fermentative enzymes; Decreased glycolytic flux with simultaneous derepression of mitochondrial genes and an increase in respiratory flux	Shift from fermentative to respiratory metabolism even with high glucose availability; elimination or strong weakening of the Crabtree effect; more efficient oxidative ATP production in “respiratory-primed” cells	[2,3,19,97,100,106]
	Ethanol production	Significantly reduced ethanol production in the early-exponential phase; Decreased pyruvate decarboxylase (PDC) activity	Redirection of carbon flux from fermentation products (ethanol, glycerol) into increased ATP production per unit of sugar consumed	[2,97,99,100,104,105,106]
	Pyruvate metabolism and TCA cycle flux	Intracellular accumulation of pyruvate with simultaneous increase in activity of mitochondrial ATPase, electron transport chain components and TCA cycle enzymes (e.g., Cit1, Idh1/2)	Enhanced mitochondrial pyruvate utilization; the lack of strong G6P-dependent feedback inhibition of alternative hexokinase (especially Glk1p) resulting in efficient respiration	[1,3,99,100,105]
	Pentose phosphate pathway (PPP)	Reduced activity of PPP enzymes (G6PD/Zwf1p, 6-PGD/Gnd1p and Gnd2p) despite an increase in the substrate pool (G6P) and upregulation of *ZWF1* transcription; Reduced NADPH consumption for anabolic processes	Reduced cellular biosynthetic burden; redirection of PPP (e.g., ribose-5-phosphate) products towards de novo nucleotide (ATP) synthesis instead of macromolecule (protein/lipid) biosynthesis	[1,3]
	Metabolic flux distribution	Global carbon flux redistribution; Decrease in carbon flux through glycolytic and fermentative enzymes (e.g., Gpm1, Eno2, Tdh3) with an increase in flux through the TCA cycle and oxidative phosphorylation	Global metabolic reprogramming, including mimicking the state of CR; possibility of simultaneous co-consumption of glucose with other carbon sources	[1,3,97,103,105,106]

### 4.3. Mitochondrial Bioenergetics and Redox Homeostasis in the Absence of Hxk2p

The deletion of the *HXK2* gene causes a relevant shift toward oxidative metabolism, effectively attenuating the Crabtree effect even in high-glucose conditions [3,96,97,98,100,103,104]. A major hallmark of this shift is a substantial increase in mitochondrial activity, manifested by a more developed mitochondrial network, increased mitochondrial membrane potential (MMP), increased mitochondrial ATPase activity, increased oxygen uptake, and pronounced derepression of genes involved in the TCA, ETC, and components of the mitochondrial translation machinery [1,2,3,100,103,104,105,106,113]. Consequently, cells of the Δ*hxk2* strain exhibit substantially elevated ATP levels in comparison to the WT strain, 50–70% higher when grown on glucose and up to 2.5-fold higher when grown on fructose. This high-energy generation in the case of the Δ*hxk2* strain is further reflected in a significantly elevated ATP/ADP ratio, confirming a reorganization of the cellular energy economy and a global redistribution of metabolic flux toward respiration. Importantly, these high ATP levels and ATP/ADP ratio are maintained regardless of external glucose concentration, making the cells of the Δ*hxk2* strain “respiratory-primed”, which also limits changes that occur during diauxic shifts [1,3,101] (Table 2). However, an increase in ATP is not solely due to higher mitochondrial activity but also results from changes in metabolic economy and reorganization of intracellular pathways, as shown by the higher ATP values in Δ*hxk2* rho^0^ cells compared to WT rho^0^ cells [3]. Despite the enhancement of respiratory flux, the cells of the Δ*hxk2* strain paradoxically generate notably lower levels of reactive oxygen species (ROS), independently of the external glucose concentration and carbon source used. This phenomenon can be explained by a “mitohormesis” effect, where enhanced mitochondrial metabolism improves the “sealing” of the respiratory chain, preventing electron leakage and subsequent oxidative damage [1,2,3,114]. At the pyridine cofactor level, the Δ*hxk2* strain has a lowered NADP+/NADPH ratio and relatively stable NADPH content, among others caused by a global reduction in cellular biosynthesis and reduced NADPH consumption for anabolic processes [1,2,3,73,102]. Moreover, reduced cAMP/PKA signaling in these cells further strengthens the oxidative stress response by activating transcription factors like Msn2/4p and increasing the expression of antioxidant defence genes dependent on them [1,3,14,102,115]. The absence of Hxk2p may also favor the opening of mitochondrial VDAC channels, potentially enhancing substrate delivery to mitochondria and improving the efficiency of respiratory metabolism [1,116]. Together, these pleiotropic adaptations in energy and redox metabolism generate a stable, stress-resistant cellular state that mimics CR conditions and may extend the reproductive potential of the cell (Table 2).

**Table 2:** Mitochondrial bioenergetics and redox homeostasis in the absence of Hxk2p.

Functional Level/ Effect Category	Parameter	Effect of *HXK2* Gene Deletion	Physiological Consequence	References
**Mitochondrial** **activity**	Mitochondrial membrane potential (MMP)	Increased MMP; Higher and comparable MMP regardless of glucose concentration; Cultivation on fructose further enhances MMP	Increased respiratory activity; persistent activation of oxidative metabolism despite the availability of sugars	[1,3]
	Mitochondrial morphology	A more developed mitochondrial network compared to the WT strain	Increased respiratory capacity and support for enhanced oxidative catabolism; altered adaptation to environmental changes and metabolic stress	[1,2,3]
	Respiratory activity	Derepression of respiratory genes (e.g., *CIT1*, *IDH1/2*); Global upregulation of genes involved in respiration and components of the mitochondrial translation machinery; Increased oxygen uptake (JO2); Significantly higher basal respiration than in the WT strain	Enhanced oxidative metabolism and ATP production via OXPHOS; systemic reprogramming of the proteome to aerobic energy production; attenuated Crabtree effect; increased mitochondrial respiration; increased sensitivity to respiratory inhibitors (e.g., antimycin A); increased metabolic efficiency and higher ATP yield per unit of carbon	[1,3,100,103,104,105,106,113]
	Mitochondrial H^+^-ATPase activity	Increased mitochondrial ATP synthase activity even from the early phase of growth on glucose	Shortening the time of metabolic adaptation during the diauxic transition; Δ*hxk2* cells are respiratory-primed; higher ATP yield per unit of carbon	[100]
**Energy** **metabolism**	ATP level	Significantly higher ATP level (increased by 50–70% on glucose and increased 2.5-fold on fructose compared to the WT strain); Higher and comparable levels of ATP regardless of glucose concentration; Higher level of ATP in Δ*hxk2* rho^0^ (respiratory-deficient) than WT rho^0^ yeast cells	Redistribution of metabolic flux towards respiration; high energy levels regardless of conditions; Δ*hxk2* cells are respiratory-primed; changes in metabolic economy and reorganization of intracellular pathways (the increase in ATP is not solely due to higher mitochondrial activity)	[1,3,101]
	Diauxic shift ATP level	No significant changes in ATP level after diauxic shift; ATP level in the WT strain increased after glucose depletion to the level observed in the Δ*hxk2* strain already in the exponential phase	Redistribution of metabolic flow towards respiration; high energy levels regardless of conditions; eliminating the rapid energy changes typical for changing carbon sources in WT cells	[3]
	ATP/ADP ratio	Similar to the WT strain level of ADP; Higher ADP–ATP pool; Significantly higher ATP/ADP ratio (2-fold higher on glucose and 3-fold higher on fructose compared to WT strain)	High cytosolic ATP/ADP ratio inhibiting glycolysis and promoting respiration; potential opening of mitochondrial VDAC channels in the absence of Hxk2p, facilitating substrate flux into mitochondria; redistribution of metabolic flux towards respiration; changes in metabolic economy and reorganization of intracellular pathways	[1,116]
	Crabtree effect	Attenuation of the Crabtree effect; “Crabtree-diminished” phenotype; Absence of immediate ethanol production following a glucose pulse (delayed short-term Crabtree effect)	Reduced reliance on fermentation metabolism even in glucose-rich conditions	[1,3,97,98,100,103,104]
**Redox** **metabolism**	Reactive oxygen species (ROS) generation	Significantly lower intracellular ROS content (both DHET and H_2_DCF-DA assays); Consistently reduced ROS generation levels, independent of glucose concentration and carbon source type (glucose or fructose)	Reduced cellular oxidative burden; improved physiological homeostasis; enhanced mitochondrial metabolism (mitohormesis effect); improved “sealing” of the respiratory chain, preventing electron leakage and consequently reducing ROS generation despite enhanced respiration	[1,2,3]
	Vulnerability to respiratory failure conditions	A greater decline in ATP content and a rapid increase in ROS generation under conditions impairing mitochondrial activity (e.g., antimycin A and oligomycin)	High dependence of Δ*hxk2* cells on mitochondrial energy production; high sensitivity to stress conditions impairing respiratory function	[1]
	Pyridine nucleotide cofactors (NAD(P)H) balance	Relatively stable NADPH level; Decreased NADP^+^ level; Significantly lower NADP^+^/NADPH ratio compared to the WT strain; Altered or reduced activity of PPP enzymes (G6PD/Zwf1p, 6-PGD/Gnd1p and Gnd2p) despite an increase in the G6P substrate pool; Redistribution of NADH production between the cytosol (reduced glycolysis) and mitochondria (active TCA cycle and respiration)	Reduced NADPH consumption for anabolic processes (biosynthesis of macromolecules); potentially limited substrate availability for NADPH oxidase (Yno1p), likely contributing to reduced non-mitochondrial ROS generation and stabilization of the cellular redox balance; altered NAD^+^ availability, likely influencing the activity of sirtuins and nuclear transcription factors; remodelling of organelle-specific redox potential distribution	[1,2,53,73,102,117]
	Glutathione metabolism	Reduced glutathione (GSH) regeneration efficiency potentially affected by altered NADPH; Reduced glutathione levels associated with pharmacological inhibition of Hxk2p (e.g., by 3-BP)	Coupling between PPP activity and cell detoxification capacity; the strain lacking the *HXK2* gene may show less GSH dependence due to low ROS levels	[118,119,120]
	Oxidative stress response	Reduced cAMP/PKA signaling resulting in activation of Msn2/4 transcription factors and STRE-mediated stress responses	Activation of defense mechanisms and gene expression of antioxidant enzymes (e.g., catalases, superoxide dismutases); altered resistance to oxidative stress and environmental stressors	[1,3,14,102,115]

### 4.4. Attenuated Biosynthetic Capacity, Enhanced Proteostasis, and Cell Size Regulation Following HXK2 Deletion

The deletion of the *HXK2* gene triggers fundamental changes in metabolic economy and a shift in cellular resource allocation, prioritizing respiratory metabolism, cell maintenance, and stress resistance over rapid proliferation and biomass growth. This metabolic reorganization is characterized by a significant reduction in the biosynthetic capacity of the cells, primarily driven by an altered metabolic flux within the glycolysis–respiration–PPP triangle. The Δ*hxk2* strain exhibits altered activity of PPP enzymes, increased levels of G6P, and changes in the NADP(H) pool, which limit the availability of PPP-derived products for the biosynthesis of macromolecules and redirect them toward nucleotide (ATP) synthesis [1,3,105,106]. This redirection of metabolic flux is accompanied by changes at the proteomic level, where a downregulation of proteins involved in ribosome biogenesis and the biosynthesis of amino acids is observed. The consequences of this are manifested by significantly lower total cellular protein content, reduced cell dry weight, decreased growth rate of the cell population, and reduced overall biosynthetic capacity of the cell, regardless of extracellular glucose concentrations [1,2,3,99,100,103,105,111,121]. The reduction of cellular biosynthetic capacity is closely coupled to cell size regulation, a key aspect of cellular physiology and morphology, and is also, as shown by current studies, an important determinant of individual cell proliferation capacity, as postulated by the hypertrophy hypothesis. According to this theory, the increase in cell size that occurs in subsequent reproductive cycles leads to a size that prevents further proliferation. This increase in cell size may be further accelerated by environmental conditions, e.g., exposure to high amounts of nutrients (calorie excess—CE) [2,3,23,122,123,124,125,126,127]. The cells of the Δ*hxk2* strain exhibit small cell size and reduced cell growth dynamics, regardless of the external glucose concentration and carbon source type. This effectively protects the Δ*hxk2* strain from CE-induced cell enlargement and the accompanying decline in cell reproductive potential [1,2,3,23,121,128]. Furthermore, the reduced biosynthetic capacity and anabolic burden are also connected with improved proteostasis and protein quality control. The deletion of the *HXK2* gene and absence of Hxk2p result in significantly increased proteasome activity (especially chymotrypsin and caspase-like activity). Additionally, increased proteasome activity enhances the degradation of the Mig1p repressor, leading to the induction of respiratory metabolism, and high levels of ATP support energy-intensive repair and degradation processes [3,101,102]. Consequently, this enhanced proteasomal activity, combined with lowered translation and reduced cellular protein content, enables the efficient removal of damaged or misfolded proteins, minimizes proteotoxic stress, improves protein turnover, and promotes amino acid recycling [1,3,101,102,129]. Ultimately, the synergy between attenuated biosynthetic capacity, cell size regulation, and enhanced proteostasis (Table 3) emphasizes the systemic nature of the enhanced cell reproductive potential in the Δ*hxk2* strain and explains metabolic changes characteristic of CR.

**Table 3:** Pleiotropic consequences of *HXK2* gene deletion for cellular biosynthesis, proteostasis, and cell proliferation capacity.

Functional Level/ Effect Category	Parameter	Effect of *HXK2* Gene Deletion	Physiological Consequence	References
**Biosynthetic** **capacity**	PPP-derived biosynthesis/ PPP flux	Reduced activity of PPP enzymes (G6PD/Zwf1p, 6-PGD/Gnd1p and Gnd2p); Increased G6P level; Change in NADP(H) pool	Altered formation of NADPH and biosynthetic precursors; reduced NADPH consumption for anabolic purposes; shift of metabolic flux towards respiration and oxidative ATP generation; redirection of PPP (ribose-5-phosphate) products towards nucleotide (ATP) synthesis instead of macromolecule biosynthesis	[1,3]
	Amino acid biosynthesis	Downregulation of proteins involved in the synthesis of amino acids, especially branched-chain amino acids (leucine, valine, isoleucine), arginine and histidine	Limited availability of intracellular building blocks; slower translation processes; reduced metabolic burden; slower growth; carbon/energy resource conservation	[105]
	Protein content	Significantly lower total cell protein content (pg/cell); Remaining consistently low cell protein content regardless of glucose concentration or carbon source type	Reduced anabolic burden; lower ATP consumption for protein biosynthesis; better proteostasis and protein quality control	[1,3]
	Cell dry weight	Significantly reduced dry cell mass compared to the WT strain; Remaining consistently low cell dry weight regardless of glucose concentration	Reduced biosynthetic capacity; reduced anabolic burden; altered cellular composition and limited accumulation of storage/building materials; phenotype mimicking caloric restriction	[3]
	Growth rate and growth kinetics	Decreased growth rate (μ) in the exponential phase; altered population growth kinetics; Slower increase in optical density (OD_600_) and prolonged time to plateau; Despite a slower growth rate, reaching a final cell population density (carrying capacity) comparable to or higher than that of the WT strain	Metabolic trade-off—slower proliferation and reduced biosynthetic burden (additionally preventing hypertrophy) allow for increased cell reproductive potential/proliferation capacity	[1,2,3,99,100,103,111,121]
**Cell size** **regulation**	Cell size	Significantly smaller average cell size, regardless of carbon source type; The Δ*hxk2* strain exhibits some of the smallest cell sizes among various yeast strains, including those with impaired glucose signaling	Small cell size and slower biosynthetic processes supporting the maintenance of physiological homeostasis; protection against calorie excess (CE)-induced decline in cell reproductive potential	[2,3,121,128]
	Cell growth vs. cell reproduction balance	Smaller cell size and slower cell growth rate, yet increased cell reproductive potential and an extended period of cell proliferation	Reduced cell volume growth dynamics, associated with maintenance of cell proliferation capacity (evidence supporting the hypertrophy hypothesis); reduction of coordination disorders between cell size and cell cycle	[1,2,3,23,122]
	Carbon source availability-dependent changes in cell size	Small mean cell size regardless of glucose concentration or carbon source type; Lack of sensitivity to extracellular glucose concentration (unlike the WT strain, mutant Δ*hxk2* maintains a constant small cell size); Fructose-grown cells exhibit even more pronounced limitations of biosynthetic capacity and smaller cell size relative to glucose-grown cells	Reduced dependence on cell size regulation in environmental signals; intracellular glucose levels and associated signaling pathways highlighted as key determinants of cell size regulation; prevention of excessive cell enlargement and protection against CE-induced hypertrophy; physiological and morphological adaptation to a CR-like state; protection against metabolic dysfunction and the negative effects of glucose excess	[1,2,3]
	Biosynthesis–cell size coupling	Strong correlation between reduced protein content (pg/cell), low cell dry weight, and smaller cell size; Decreased flux through the PPP, limiting the availability of building blocks for macromolecular synthesis; Lower usage of NADPH	Lower amount of intracellular macromolecules (mainly proteins) translating directly into smaller physical cell volume; reduction of anabolic burden; lower biosynthetic efficiency, and smaller cell sizes as important aspects accompanying the energetic and metabolic changes observed in the *HXK2* deletion mutant strain	[1,3,23]
**Proteostasis**	Proteasomal activity	Increased proteasome activity (especially chymotrypsin and caspase-like activity) regardless of glucose availability; Increased proteasome-dependent degradation of the Mig1p repressor	Improved protein turnover and amino acid recycling; improved proteostasis and potentially improved removal of damaged proteins; induction of respiratory metabolism through Mig1p degradation	[3,101,102]
	Protein turnover and quality control	Reduced biosynthetic capacity; Increased proteasomal activity; High and stable ATP levels in the Δ*hxk2* strain, supporting energy-intensive repair and degradation processes	Increased efficiency of removal of non-functional, damaged and aggregated proteins via the UPS (ubiquitin–proteasome system); prevention of the accumulation of protein aggregates; better tolerance to proteotoxic stress; improvement of the physiological capabilities of cells	[3,101,102,129]
	Proteome remodeling	Global remodeling of protein composition; Downregulation of glycolytic enzymes and proteins associated with biosynthetic pathways, including amino acid biosynthesis, upregulation of mitochondrial proteins, TCA cycle enzymes, and mitochondrial translation machinery	Systemic adaptation to a “respiratory-primed” state; shifting the cell’s priorities from fast proliferation to energy efficiency and defense mechanisms characteristic of caloric restriction	[1,102,105,106]
	Biosynthetic burden	Lower total cellular protein content; Decreased translation and ribosome biogenesis resulting from decreased activity of the cAMP/PKA pathway	Reduced anabolic burden; reduced risk of protein misfolding due to lower translation rates; reduced energetic expenditure on biosynthesis, allowing resource allocation toward proteostasis and lifespan extension	[1,3,101]
**Reproductive lifespan/** **Cell proliferation capacity**	Reproductive potential	Increased mean and maximal cell reproductive potential (increased number of daughter cells); Mean reproductive potential of the cells increased approximately 30–50%; Increased cell reproductive potential regardless of glucose concentration or carbon source type	Extended replicative lifespan of a cell; increased cell proliferation capacity	[1,3,93,94,95,96,102]
	Calorie restriction (CR) mimicry	The *HXK2* gene deletion generates a CR-like transcriptional and metabolic profile even under glucose-rich conditions; ∆*hxk2* mimics the CR phenotype	Imitation of nutrient-limited conditions; sustained activation of protective mechanisms, respiratory metabolism, mitohormesis, and decreased cellular biosynthesis; improved physiological state and promotion of increased cell proliferation capacity	[1,2,3,93,94,95,96,101,102]
	Nutrient-induced changes in reproductive potential	Cell reproductive potential unaffected by glucose concentration; Slight effect of fructose on cell reproductive potential; Protection against the decline in cell reproductive potential induced by glucose excess	Lifespan extension mechanism; protection from calorie excess (CE)-induced lifespan decline; resistance to metabolic stress induced by CE; CR-mimetic phenotype	[1,3,23]
	Metabolism–lifespan link	Close correlation of lifespan extension with remodeling of the central carbon flux, reduced anabolic burden, and smaller cell size	Strong coupling between metabolism and reproduction; system-level regulation of proliferation and aging; cell proliferation capacity as a trade-off between biosynthesis efficiency, growth, cell size, proteome quality, and metabolic changes	[1,2,3,23]

### 4.5. Systemic Metabolic Adaptation as a Driver of Increased Cell Proliferation Capacity in Yeast

The deletion of *HXK2* markedly increases cell reproductive potential (extends the replicative lifespan (RLS)) of *S. cerevisiae*. The Δ*hxk2* strain shows increases in both mean and maximum cell reproductive potential, with mean reproductive potential increased by approximately 30–50%, regardless of glucose concentration or carbon source type [1,3,93,94,95,96,102]. This increased cell reproductive potential is primarily attributed to the fact that the Δ*hxk2* strain mimics the CR phenotype and exhibits a CR-like transcriptional and metabolic profile even under glucose-rich conditions [1,2,3,101]. The basis of this increased cell proliferation capacity is a system-level metabolic remodeling that promotes respiratory metabolism but simultaneously reorganizes intracellular pathways, leading to reduced biosynthetic activity, smaller cell size, improved proteostasis, and enhanced stress resistance. An important element connected with increasing the reproductive potential of cells is the regulation of cell size and reduction of anabolic burden. Therefore, unlike wild-type cells that undergo glucose-induced cell enlargement, the Δ*hxk2* strain maintains a constant small cell size. Moreover, the increased cell reproductive potential is supported by enhanced proteostasis, characterized by elevated proteasomal activity and a reduced anabolic burden, which may minimize proteotoxic stress. These effects, maintained even under CE conditions, shield the Δ*hxk2* strain against the reproductive potential decline observed in the wild-type strain [1,2,3,23,101,122]. These adaptations result in a slower growth rate; however, the Δ*hxk2* strain achieves a comparable or higher final cell population density (carrying capacity) than the wild-type strain. Consequently, the synergistic interplay between enhanced respiratory metabolism, altered cellular bioenergetics, reduced biosynthetic activity, and improved proteostasis and protein turnover, noted in the absence of Hxk2p, may represent a central determinant of the maintenance of cellular proliferation capacity (Table 3).

**Table 4:** Gene expression regulation and Hxk2 subcellular dynamics.

Functional Level/ Effect Category	Parameter	Effect of *HXK2* Gene Deletion	Physiological Consequence	References
**Gene expression** **regulation**	Global transcription remodeling	Systemic reprogramming of the transcriptome; Significant derepression of genes for aerobic metabolism and alternative carbon sources, concomitantly reduced expression of glycolytic and biosynthetic genes	Transcription profile mimicking the profile of cells growing on non-fermentable sources and under CR conditions; expanded capacity to utilize different carbon sources and orientation towards efficient energy use	[1,3,21,105,106,107,130]
	Transcription factor activity	Disintegration of the nuclear Hxk2–Mig1/2 repressor complex, resulting in Mig1p export from the nucleus and loss of transcriptional repression; Reduced cAMP/PKA signaling leading to derepression of Cat8p (nonfermentative metabolism), Hap4p (mitochondrial biogenesis), and Msn2/4p (stress response) regulatory pathways	Relief of catabolic repression; coordinated activation of the respiratory and stress response genes normally inhibited by glucose	[14,19,21,105]
	Genes with increased expression (Upregulated/Derepressed)	Alternative sugar metabolism and classical derepression: *SUC2* (invertase); galactose metabolism genes: *GAL1*, *GAL2*, *GAL3*, *GAL7*, *GAL10*; maltose metabolism genes: *MAL31*, *MAL32;* Compensatory sugar phosphorylation system: *HXK1* (hexokinase 1), *GLK1* (glucokinase), *EMI2* (glucose-limited hexokinase); Moderate- and high-affinity hexose transporters: *HXT2*, *HXT4*, *HXT6*, *HXT7*; TCA cycle and respiratory chain genes: *CIT1* (citrate synthase), *IDH1*, *IDH2* (isocitrate dehydrogenase), *KGD1*, *KGD2* (α-ketoglutarate dehydrogenase), *LSC1*, *LSC2* (succinyl-CoA synthetase), *SDH1*, *SDH2*, *SDH4* (succinate dehydrogenase), *COX2* (cytochrome c oxidase), *ATP3*, *ATP17* (ATP synthase); Other metabolic pathway genes: *ALD7*, *ACS1* (acetate metabolism), *ADH2* (alcohol dehydrogenase II), *ICL1*, *MLS1* (glyoxylate cycle), *FBP1*, *PCK1* (gluconeogenesis), *ZWF1* (glucose-6-phosphate dehydrogenase), *RIM4* (meiosis regulator, co-regulated by Ash1p)	Co-consumption of different sugars; absence of an adaptation phase upon carbon source switching; attenuation/elimination of the Crabtree effect	[1,19,20,21,84,98,99,100,103,104,105,106]
	Genes with decreased expression (Downregulated)	Low-affinity hexose transporters: *HXT1*, *HXT3*; Glycolysis: *TDH3* (GAPDH), *PGK1* (phosphoglycerate kinase), *GPM1* (phosphoglycerate mutase), *ENO2* (enolase 2), *CDC19* (pyruvate kinase), *PDC1* (pyruvate decarboxylase); Amino acid biosynthesis genes: *HIS3*, *HIS4*, *HIS5*, *HIS7* (histidine synthesis), *LEU1*, *LEU4* (leucine synthesis), *VAL1* (valine synthesis), *ILE1* (isoleucine synthesis), *ARG8* (arginine synthesis), *GLT1*, *GLN1* (glutamate/glutamine synthesis), *SER33*, *MET1*, *MET2*, *MET16*; Ribosome biogenesis: RPs (ribosomal proteins) genes (effect of reduced cAMP/PKA signaling) Other cellular processes: *BUD20*, *SAN1*, *FET4*	Reduced anabolic burden and conservation of energy (ATP) and carbon resources, promoting smaller cell size and enhanced proteostasis	[1,21,99,100,103,105,106]
**Intracellular** **localisation and** **PTM regulation**	Nuclear role & Moonlighting	Loss of nuclear function of Hxk2p (normally constitutes approximately 15% of the total cellular pool); Impaired formation of the Hxk2p–Mig1p repressor complex; Disrupted interaction with accessory proteins (e.g., Med8p, Tup1p) and loss of transcriptional repression of target genes; Predominant cytoplasmic localization of Mig1p, even under high-glucose conditions; Loss of non-catalytic (“moonlighting”) regulatory functions of Hxk2p, not efficiently compensated by Hxk1p	Removal of nuclear glucose repression; reduced integration between carbon metabolism and transcriptional control; altered metabolic control of nuclear gene expression	[20,21,80,83,86,88,89,130]
	Hexokinase post-translational modifications	Elimination of the Hxk2p-specific PTM sites (Hxk2p-specific post-translational modifications absent in the paralog Hxk1p; Ser15 phosphorylation site, the primary target of Tda1p kinase, and unique Lys13 sumoylation/methylation site); Altered glucose-dependent structural switching controlling regulatory functions of Hxk2p (PTM regulates the dimer–monomer transition of Hxk2p; cells show a dynamic equilibrium between the unphosphorylated dimeric form and the Ser15-phosphorylated monomeric form of Hxk2p; the monomeric form of Hxk2p shows nuclear accumulation)	Disrupted conformational and oligomeric hexokinase regulation, preventing efficient DNA-associated repression complex formation; disruption of glucose-dependent nuclear repression mechanisms	[19,29,80,82,86,130]
	Snf1 recruitment and activation	Removal of Hxk2p-mediated coupling between Snf1p kinase, Reg1p phosphatase, and Mig1p repressor; Lack of recruitment of Mig1p/Snf1p regulatory complex to target gene promoters (e.g., *SUC2*); Persistent Snf1p activity, even in the presence of excess glucose	Disintegration of the nuclear repression complex; persistent phosphorylation and export of Mig1p to the cytoplasm; impaired gene silencing in the presence of glucose; perception of a cellular energy deficit signal despite sugar availability, promoting a shift toward aerobic metabolism and activation of CR-like mechanisms	[19,20,21,86,102,130,131]
	Hexokinase structural and conformational sensor mechanism	Loss of the glucose-induced conformational change; Absence of transition between open and closed conformation states; Elimination of the Hxk2p-specific decapeptide (Lys6–Met15) regulatory motif required for Mig1p binding	Disruption of the structural signal required for Hxk2p–Mig1p interaction; disruption of glucose-dependent gene repression mechanisms (e.g., *SUC2* repression), even if hexokinase catalytic activity is maintained	[19,132,133,134]
	Chromatin stability	Reduced chromatin instability and loss of gene silencing (e.g., at the HML locus) following *HXK2* gene deletion	Improved genome integrity; more stable chromatin structure supporting extended replicative lifespan and improved cellular homeostasis	[135]

### 4.6. Global Transcriptional Remodeling and Removal of Glucose-Mediated Gene Repression in the Absence of Hxk2p

The deletion of the *HXK2* gene results in the disruption of the CCR mechanism in *S. cerevisiae.* This is primarily due to the disassembly of the nuclear Hxk2p–Mig1p repressor complex, which leads to the export of the Mig1p repressor to the cytoplasm even when glucose is abundant. Therefore, the yeast transcriptome undergoes systemic reprogramming, where the expression of genes of respiratory and alternative carbon source metabolism is derepressed, while the expression of glycolytic and biosynthetic genes is downregulated [1,3,14,19,21,80,84,99,105,106] (detailed list of genes with altered expression—Table 4). Among the most highly upregulated genes are: (i) alternative sugar utilization genes, such as *SUC2* (encoding invertase), galactose metabolism (*GAL1*, *GAL7*, *GAL10*), and maltose metabolism (*MAL31*, *MAL32*) genes; (ii) genes involved in mitochondrial biogenesis and aerobic metabolism, including TCA cycle enzymes (*CIT1*, *IDH1/2*, *SDH1/2/4*) and respiratory chain components (*COX2*, *NDI1*, *ATP3*); (iii) genes of hexokinase isoenzymes necessary for compensatory sugar phosphorylation reactions, i.e., *HXK1* (hexokinase 1), *GLK1* (glucokinase), *EMI2* (glucose-limited hexokinase); (iv) moderate- and high-affinity hexose transporter genes like *HXT2*, *HXT4*, *HXT6* and *HXT7*; (v) other metabolic pathway genes like *ACS1*, *ADH2*, *ZWF1,* etc. [1,19,20,21,84,98,99,100,103,104,105,106]. In turn, the most significantly downregulated genes include: (i) relevant glycolytic and fermentative genes, such as *TDH3* (GAPDH enzyme), *PGK1* (phosphoglycerate kinase), *ENO2* (enolase), and *PDC1* (pyruvate decarboxylase); (ii) low-affinity hexose transporter genes, specifically *HXT1* and *HXT3*; (iii) genes related to anabolic processes, including ribosome biogenesis (ribosomal protein [RP] genes) and amino acid biosynthesis (e.g., *HIS3*, *HIS4*, *LEU1*, *VAL1*, *ARG8*, *GLT1*, *MET1*, *MET2*) genes [1,21,99,100,103,105,106]. Physiological consequences of this transcriptional remodeling are a reduction in the cellular biosynthetic burden, small cell size, an alteration in glucose uptake capacity, a shift to oxidative bioenergetics, and the elimination of lag phases during nutrient transitions. Ultimately, this transcriptomic profile mimics the profile of cells cultured under CR conditions and plays an important role in increasing cell reproductive potential in the Δ*hxk2* strain.

### 4.7. Post-Translational Regulation, Structural Dynamics, and Nucleocytoplasmic Shuttling of Hxk2p

Hxk2p is a moonlighting protein that shuttles between the cytoplasm and the nucleus to integrate metabolic flux with global transcriptional control [20,21,80,83,86,88,89,130] (Table 4). Its intracellular distribution and regulatory functions are modulated by post-translational modifications (PTMs), primarily the phosphorylation of Ser15 (by Tda1 and Snf1 kinases) and the sumoylation or methylation of Lys13. PTMs of Hxk2p regulate its dimer–monomer transitions, where unphosphorylated Hxk2p can create the dimer form, while the Ser15-phosphorylated monomeric form can translocate to the nucleus. Beyond post-translational modifications, the dual nature of Hxk2p as a ‘moonlighting’ protein is driven by its conformational changes and physical separation of its catalytic and regulatory domains [19,132,133,134]. A non-canonical role of Hxk2p in glucose signaling is strictly dependent on a specific decapeptide motif located at the N-terminus (Lys6–Met15). Analysis of the *hxk2wrf* mutant (lacking the Lys6–Met15 motif) has demonstrated that this sequence is essential for the physical interaction between Hxk2p and the Mig1p repressor. However, its deletion does not impair hexokinase activity [133]. Structurally, Hxk2p, which is composed of a large and a small domain separated by a deep cleft containing the active site, undergoes a significant ‘induced fit’ conformational change upon glucose binding. In the absence of glucose (low-glucose conditions), the enzyme remains in an ‘open conformation’. Upon glucose binding, the large and small domains of the protein undergo movement that closes the cleft by approximately 8 Å. This ‘closed conformation’ promotes physical interaction between Hxk2p and the Mig1p repressor in the nucleus. In contrast, glucose analogues such as xylose, which lack the 6-hydroxymethyl group essential for inducing proper cleft closure, fail to promote the Hxk2p–Mig1p interaction, thereby mimicking low-glucose conditions. Therefore, the recruitment of Hxk2p to the *SUC2* repressor complex is primarily regulated by glucose-induced conformational states [19,132,134]. These findings point to the moonlighting nature of Hxk2p, in which structural signals, such as the glucose-induced conformational change, together with its specified N-terminal motif, act to coordinate the cellular response to glucose availability. Therefore, the deletion of the *HXK2* gene and the absence of Hxk2p disrupt the conformational regulation of hexokinase, prevent the efficient formation of DNA-associated repression complexes, and disrupt coupling among Snf1p kinase, Reg1p phosphatase, and the Mig1p repressor, leading to the constitutive activation of Snf1p regardless of glucose availability [19,20,21,29,80,82,86,102,130,131]. Recent studies have also shown that Hxk2p may have an impact at the epigenetic level, since *HXK2* gene deletion decreases chromatin instability and the loss of gene silencing (e.g., at the HML locus), which can improve genome integrity and support increased cell replicative potential [135].

## 5. Conclusions

**Figure 1:**
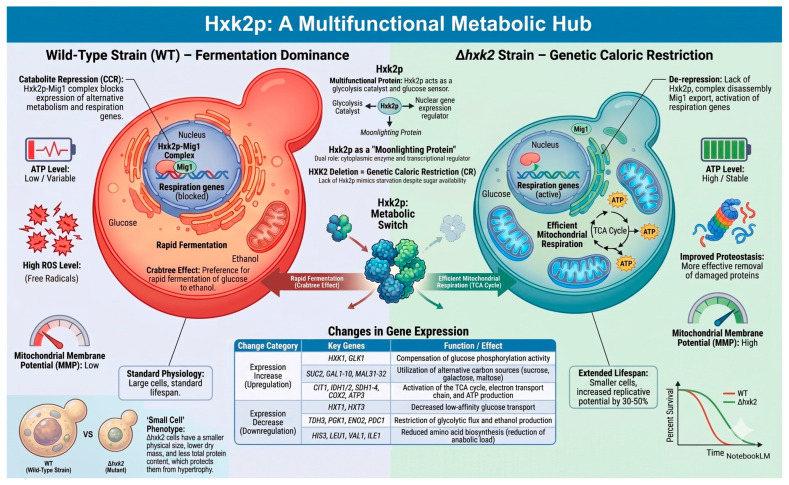
Hxk2p: Multifunctional metabolic hub in *S. cerevisiae* cells. The figure illustrates the role of the Hxk2p protein as a moonlighting protein, which functions both as a metabolic enzyme and a transcriptional regulator. The diagram shows metabolic, regulatory, and physiological differences between WT and Δ*hxk2* mutant cells. The WT strain represents standard fermentative metabolism conditions with the Crabtree effect. The Δ*hxk2* strain represents a state of genetic caloric restriction (CR), where the lack of Hxk2p mimics CR conditions despite the availability of sugar. The graphic compares these two cell types in terms of the following aspects: carbon catabolite repression (CCR), mitochondrial activity, ROS levels, ATP levels, reproductive potential, cell size, proteostasis, and global gene expression changes. Note. The graphic was created using the NotebookLM (Google LLC) application based on content prepared and verified by the authors. Gemini (Google LLC) was used to translate the text in the graphic and for technical visual refinement of the generated figure. The generated graphics were further refined by the authors through editing in graphics programs. All scientific content, labels, and interpretations were created and verified by the authors.

Hexokinase 2 (Hxk2p) functions as a multifaceted metabolic hub in *S. cerevisiae*, acting not only as the crucial glycolytic enzyme but also as an important regulator of carbon catabolite repression and an intracellular glucose sensor. The deletion of the *HXK2* gene triggers a multidirectional remodeling of cellular metabolism and physiology (Figure 1). This remodeling includes a system-level shift from a rapid fermentative mode to an energy-efficient respiratory state, characterised by the attenuation of the Crabtree effect and significantly elevated intracellular ATP levels. This is accompanied by global transcriptional remodeling, which includes derepression of mitochondrial metabolism and alternative carbon utilization genes, with simultaneous downregulation of genes concerning energetically costly biosynthetic processes. The results of this alteration are a reduction of anabolic burden and enhanced proteostasis, which lead to consistently smaller cell sizes, lowered protein biosynthesis, and enhanced protein quality control, protecting cells of the Δ*hxk2* strain from glucose-induced hypertrophy and the associated decline in cell reproductive potential. This synergy between altered bioenergetics, reduced biosynthetic and oxidative burden, and improved cell size regulation appears to be a determinant of significantly increased cell reproductive potential. At the same time, it causes the Δ*hxk2* strain to act as the genetic equivalent of calorie restriction, even in glucose-rich environments.

## Data Availability

No new data were created or analyzed in this study. Data sharing is not applicable to this article.

## Abbreviations

The following abbreviations are used in this manuscript:

ADP	Adenosine diphosphate
ATP	Adenosine triphosphate
cAMP	Cyclic adenosine monophosphate
CCM	Central carbon metabolism
CCR	Carbon catabolite repression
CE	Calorie excess
CR	Calorie restriction
DHET	Dihydroethidium
ETC	Electron transport chain
F1,6bP	Fructose-1,6-bisphosphate
G6P	Glucose-6-phosphate
GPCR	G-protein-coupled receptor
GSH	Glutathione
H_2_DCF-DA	2′,7′-dichlorodihydrofluorescein diacetate
HXKs	Hexokinases
HXTs	Hexose transporters
MMP	Mitochondrial membrane potential
mRNA	Messenger ribonucleic acid
NAD(P)H	Nicotinamide adenine dinucleotide phosphate
OXPHOS	Mitochondrial oxidative phosphorylation
PKA	Protein kinase A
PPP	Pentose phosphate pathway
PTMs	Post-translational modifications
RLS	Replicative lifespan
ROS	Reactive oxygen species
SGD	*Saccharomyces* Genome Database
STRE	Mediated stress response
TCA	Tricarboxylic acid cycle
TORC1	Target of Rapamycin Complex 1
UPS	Ubiquitin–proteasome system
VDAC	Voltage-dependent anion channel
